# Dual-negative expression of Nrf2 and NQO1 predicts superior outcomes in patients with non-small cell lung cancer

**DOI:** 10.18632/oncotarget.17403

**Published:** 2017-04-25

**Authors:** Ying-Hui Tong, Bo Zhang, You-You Yan, Yun Fan, Jia-Wen Yu, Si-Si Kong, Dan Zhang, Luo Fang, Dan Su, Neng-Ming Lin

**Affiliations:** ^1^ Laboratory of Clinical Pharmacy, Zhejiang Cancer Hospital, Hangzhou, Zhejiang 310022, China; ^2^ Laboratory of Clinical Pharmacology, Translational Medicine Research Center, Hangzhou First People's Hospital, Nanjing Medical University, Hangzhou, Zhejiang 310006, China; ^3^ Key Laboratory Diagnosis and Treatment Technology on Thoracic Oncology (Esophagus, Lung), Zhejiang Cancer Hospital, Hangzhou, Zhejiang 310022, China

**Keywords:** non-small cell lung cancer (NSCLC), NF-E2-related factor 2 (Nrf2), NAD(P)H quinone oxidoreductase-1 (NQO1), prognosis, dual-negative expression

## Abstract

Functional studies in non-small cell lung cancer (NSCLC) patients revealed that hyperactivation of the NF-E2-related factor 2 (Nrf2) pathway facilitates tumor growth. We examined the usefulness of Nrf2 and NQO1 as indicators of prognosis in NSCLC. Tumor and adjacent non-tumor tissue samples were collected from 215 NSCLC patients who had tumor resections between 2006 and 2011. Immunohistochemistry was performed to detect Nrf2 or NQO1 expression. The correlation between Nrf2 or NQO1 expression and survival outcomes was evaluated using the Kaplan-Meier method and Cox proportional hazards regression model. Levels of Nrf2 and NQO1 were elevated in tumor tissues. In particular, Nrf2 was elevated in nearly all tumor cells. NQO1 expression positively correlated with Nrf2 expression (*P* = 0.039). Nrf2 expression positively correlated with lymph node metastasis (*P* = 0.001) and negatively correlated with tumor differentiation (*P* = 0.032). As compared with either Nrf2 or NQO1 alone, dual-negative expression of Nrf2 and NQO1 was more predictive of superior overall survival (*P* = 0.020) and disease free survival (*P* = 0.037). Subgroup analyses showed that females, nonsmokers, and patients with advanced-stage NSCLC were suitable populations in which to evaluate prognosis based on Nrf2 and NQO1 co-expression. These results indicate that dual-negative expression of Nrf2 and NQO1 is predictive of a better prognosis in NSCLC patients.

## INTRODUCTION

Non-small cell lung cancer (NSCLC) accounts for 85% to 90% of total pulmonary malignancies [1], and it is the most lethal cancer worldwide [2]. Precision medicine relies on the exploration of specific biomarkers. Drugs that bind to specific biomarkers, such as EGFR, have already been widely applied in clinical settings. However, the 5-year survival of NSCLC patients is still as low as 15% [1]. Other reliable biomarkers should be considered in assisting to determine NSCLC prognosis and treatment.

NF-E2-related factor 2 (Nrf2) is a member of the Cap'n'Collar/basic leucine zipper (CNC-bZIP) transcription factor family. Functional studies showed Nrf2 is a factor in tumor initiation and progression [3]. Constitutive activation of Nrf2 provides growth advantages and confers chemo-resistance to lung cancer cells [4–6]. Suppressing Nrf2 by use of shRNA or siRNA inhibited the proliferation of NSCLC cells and increased the sensitivity of tumor cells to chemotherapeutic drugs [5, 6]. Nrf2-deficient mice showed more susceptibility to pulmonary metastasis of the mouse Lewis lung carcinoma cells [7]. Nrf2 promotes transcription of various genes, including NQO1, via the antioxidant response element (ARE) [8–10]. The functions of NQO1 include protecting against natural and exogenous quinones, maintaining endogenous antioxidants, stabilizing the p53 protein, and, at high levels, accelerating tumor progression [11]. Previous studies showed that Nrf2 or NQO1 expression was elevated in tumor tissues and correlated with the poor outcomes of patients with gastric cancer [12, 13]. We examined the expression of Nrf2 and NQO1 in Chinese NSCLC patients by immunohistochemistry (IHC) assay and investigated their prognostic significance.

## RESULTS

### Patients

A total of 215 Chinese NSCLC patients, comprising 170 males and 45 females, with ages ranging from 34 to 76 years (median 61 years), were included. The histopathology of patients included adenocarcinoma (97 patients), squamous cell carcinoma (112 patients), and other types of carcinoma (6 patients, such as adenosquamous carcinoma, large cell carcinoma, and sarcoma). Stage was classified according to TNM classification. Among all patients, 128 were at the early stage (I–II) and 87 were at the advanced stage (III–IV). In addition, 116 patients (54%) received chemotherapy (mainly platinum-based chemotherapy) and 31 (14.4%) received radiotherapy. Detailed clinico-pathological characteristics of eligible patients are listed in Table 1.

**Table 1:** Nrf2 and NQO1 expression in different clinicopathological characteristic groups

Characteristics	Total	Nrf2		*P* Value	NQO1		*P* Value
		Low, n (%)	High, n (%)		Low, n (%)	High, n (%)	
Age (y)				0.110			0.241
<Median^a^	106	28 (26.4%)	78 (73.6%)		68 (64.2%)	38 (35.8%)	
≥Median	109	40 (36.7%)	69 (63.3%)		79 (72.5%)	30 (27.5%)	
Gender				1.000			0.283
Male	170	54 (31.8%)	116 (68.2%)		111 (65.3%)	57 (34.7%)	
Female	45	14 (31.1%)	31 (68.9%)		34 (75.6%)	11 (24.4%)	
Histopathology				0.701			0.776
Adenocarcinoma	97	32 (33.0%)	65 (67.0%)		64 (66.0%)	33 (34.0%)	
Squamous cell carcinoma	112	35 (31.3%)	77 (68.7%)		79 (70.5%)	33 (29.5%)	
Others^b^	6	1 (16.7%)	5 (83.3%)		4 (66.7%)	2 (33.3%)	
Lymph node metastasis				0.001			0.232
No	91	40 (44.0%)	51 (56.0%)		67 (73.6%)	24 (26.4%)	
Yes	124	28 (22.6%)	96 (77.4%)		80 (64.5%)	44 (35.5%)	
TNM stage				0.370			0.460
I–II	128	44 (34.4%)	84 (65.6%)		90 (70.3%)	38 (29.7%)	
III–IV	87	24 (27.6%)	63 (72.4%)		57 (65.5%)	30 (34.5%)	
Differentiation				0.032			0.541
Good–moderate	101	24 (23.8%)	77 (76.2)		72 (71.3%)	29 (28.7%)	
Poor	97	37 (38.1%)	60 (61.9%)		65 (67.0%)	32 (33.0%)	
Smoking history				0.717			1.000
No	47	13 (27.7%)	34 (72.3%)		32 (68.1%)	15 (31.9%)	
Yes	142	44 (31.0%)	98 (69.0%)		98 (69.1%)	44 (30.9%)	

^a^The median age is 61 years old.

^b^Others, including adenosquamous carcinoma, large cell carcinoma, and sarcoma. Data were analyzed by the chi-square test.

### Nrf2 and NQO1 expression and correlations

**Figure 1:**
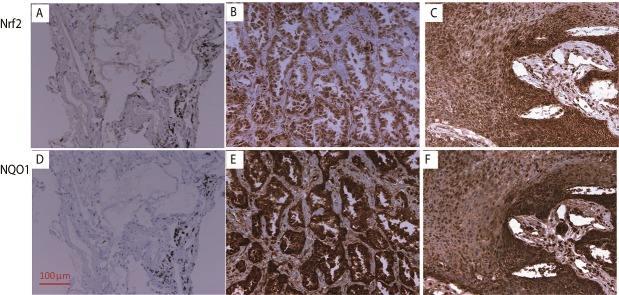
The IHC staining of Nrf2 and NQO1 (200x) The expression of Nrf2 in normal lung tissue **(A)**, adenocarcinoma **(B)**, and squamous cell carcinoma **(C)**. The expression of NQO1 expression in normal lung tissue **(D)**, adenocarcinoma **(E)**, and squamous cell carcinoma **(F)**.

Positive expressions of Nrf2 and NQO1 in tumor tissues are presented in Figure 1. Expressions in non-tumor lung tissues are also shown in Figure 1. Compared with adjacent non-tumor tissues, the expression of Nrf2 is elevated in nearly all tumor tissues. For Nrf2, there was specific staining in the nuclei of some tumor cells. High Nrf2 expression (score 9, or nucleus staining positive) was detected in 68.4% of NSCLC patients (147 of 215), although the high expression of NQO1 (score ≥ 6) was seen in 31.6% of NSCLC patients (68 of 215). NQO1 expression is correlated with Nrf2 expression (Supplementary Table 1, *P* = 0.039). More than 80% of patients (55 of 68) with high NQO1expression had high Nrf2 expression.

The differences in Nrf2 and NQO1 expression levels in different clinicopathological groups were analyzed. The clinical and pathologic characteristics and the expression levels of Nrf2 and NQO1 are summarized in Table 1. High Nrf2 expression was correlated with lymph node metastasis (77.4% vs 56.0%, *P* = 0.001) and clear differentiation (76.2% vs 61.9%, *P* = 0.032). However, the expression of NQO1 did not show any significant differences in any clinicopathological patterns.

### The role of Nrf2 and NQO1 expression in survival prediction of NSCLC patients

**Figure 2:**
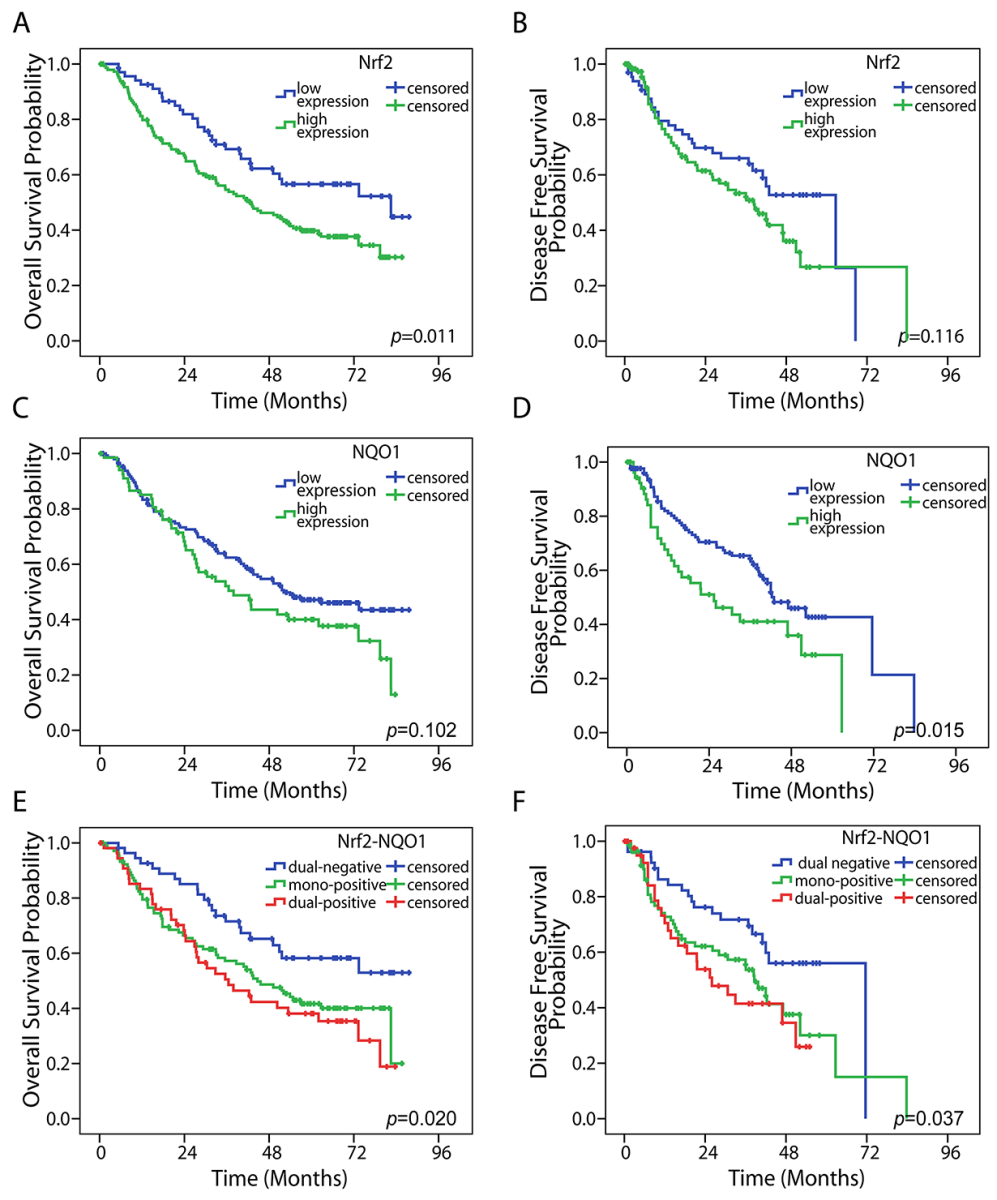
Kaplan-Meier survival analysis High Nrf2 expression was associated with a poor OS in NSCLC **(A)** (*P* = 0.011), but no significance in DFS **(B)** (*P* = 0.116). High NQO1 expression was correlated with a poor OS, but there was no significance **(C)** (*P* = 0.102). High NQO1 expression was correlated with a poor DFS **(D)** (*P* = 0.015). Dual-negative expression of Nrf2 and NQO1 was associated with both longer OS **(E)** (*P* = 0.020) and poor DFS **(F)** (*P* = 0.037). The statistical significance was assessed by use of the log-rank test.

The Kaplan-Meier method was used to analyze the correlations between Nrf2 or NQO1 expression and patients’ survival outcomes. The results of survival analysis are presented in Figure 2. Nrf2 is a predictive factor for overall survival (OS) (*P* = 0.011), and NQO1 is a predictive factor for disease free survival (DFS) (*P* = 0.015). Although high expression of Nrf2 or NQO1tended to indicated a poor DFS or OS, respectively, there was no statistical significance (*P* = 0.116 and *P* = 0.102, respectively). We combined Nrf2 and NQO1 as a co-factor for survival analysis (Figure 2). Patients were thus classified into three groups according to the IHC results: dual-positive (high expression levels of both Nrf2 and NQO1, n = 55), mono-positive (high expression levels of either Nrf2 or NQO1, n = 105), and dual-negative (Nrf2 low expression and NQO1 low expression, n = 55). Results revealed that dual-negative expression of Nrf2 and NQO1 indicated better OS (*P* = 0.020) and DFS (*P* = 0.037) (Figure 2).

To further elucidate factors that influence long-term survival, a series of Cox analyses was used for all variables listed in Table 2. We also used Nrf2 and NQO1 expressions as a co-factor. Univariate analysis demonstrated that Nrf2-NQO1 expression, lymph node metastasis status, and TNM stage were correlated with DFS and OS in patients with NSCLC (Table 2). In addition, we performed a multivariate analysis of these three factors as an independent prognostic marker. Nrf2-NQO1 expression and the tumor stage were considered as independent prognostic factors.

**Table 2:** Univariate and multivariate Cox proportional hazards analyses for disease-free survival and overall survival

	Disease-Free Survival		Overall Survival	
	HR (95%CI)	*P* Value	HR (95%CI)	*P* Value
**Univariate**				
Nrf2–NQO1	1.439 (1.079–1.920)	0.013	1.410 (1.096–1.814)	0.007
Age (y)	0.999 (0.653–1.529)	0.996	1.378 (0.955–1.990)	0.085
Gender	0.867 (0.508–1.478)	0.595	0.628 (0.380–1.039)	0.056
Histopathology	1.429 (0.981–2.081)	0.063	1.113 (0.801–1.546)	0.525
Lymph node metastasis	1.751 (1.125–2.727)	0.013	2.175 (1.462–3.237)	0.000
TNM stage	1.926 (1.251–2.963)	0.003	2.543 (1.761–3.673)	0.000
Grade group	1.231 (0.786–1.927)	0.364	1.227 (0.835–1.804)	0.297
Smoking history	0.867 (0.523–1.438)	0.580	1.196 (0.752–1.902)	0.441
**Multivariate**				
Nrf2–NQO1	1.393 (1.033–1.879)	0.030	1.364 (1.053–1.767)	0.019
Lymph node metastases	1.176 (0.680–2.033)	0.563	1.285 (0.786–2.100)	0.318
TNM stage	1.738 (1.032–2.928)	0.038	2.209 (1.412–3.457)	0.001

CI: confidence interval. Log-rank test was used to evaluate the statistical significance of variables in survival distribution. Multivariate analysis was performed by use of the Cox proportional hazard regression analysis.

### Subgroup analyses

**Figure 3:**
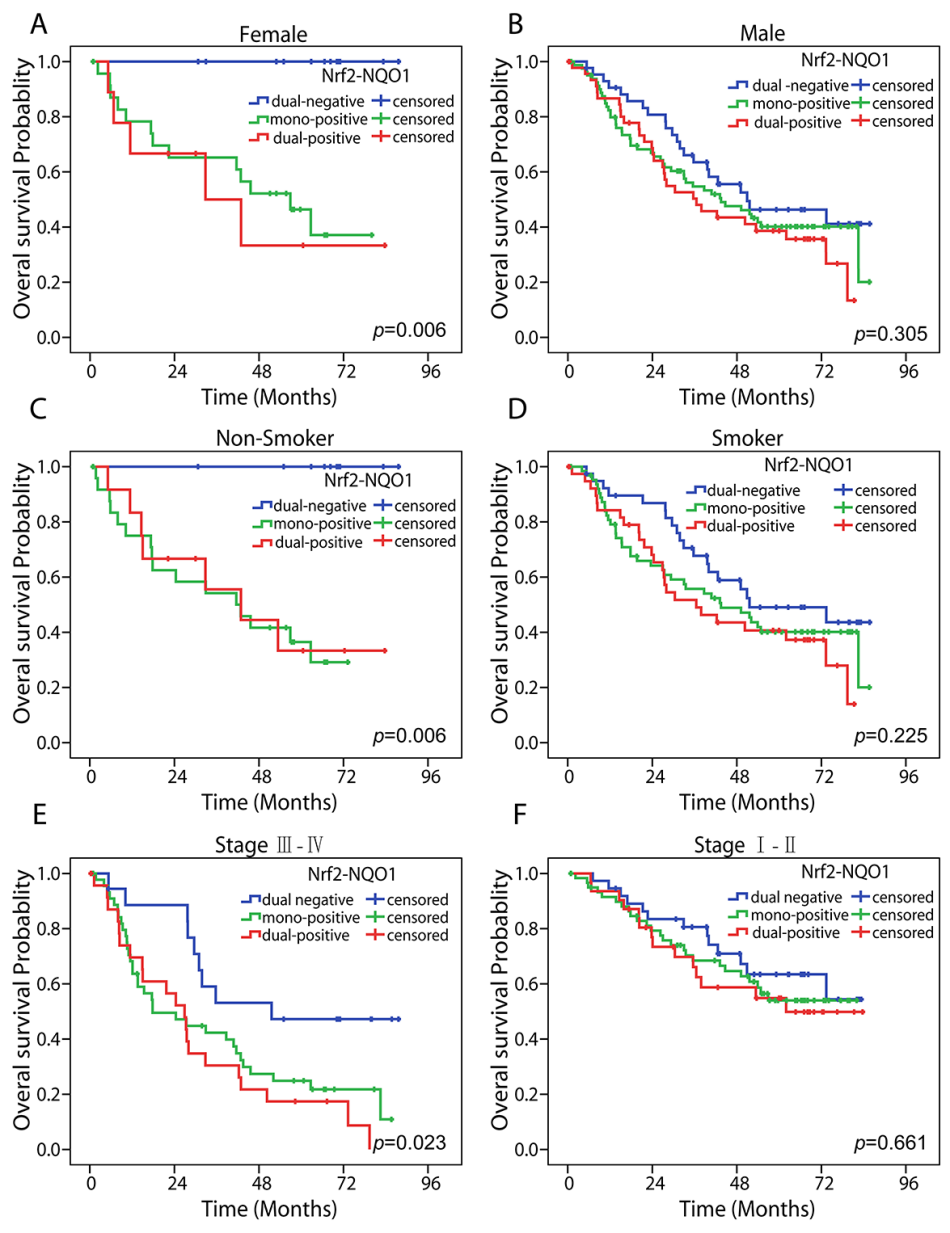
Subgroup analyses of Nrf2-NQO1 in predicting OS Kaplan-Meier survival analysis estimates the predictive role of Nrf2-NQO1 by overall survival rates in female **(A)**, male **(B)**, nonsmoker **(C)**, smoker **(D)**, advanced-stage NSCLC **(E)**, and early stage NSCLC **(F)** populations. The statistical significance was assessed using the log-rank test.

Females, nonsmokers, and patients with advanced-stage NSCLC might be suitable subpopulations for predicting prognosis by Nrf2 and NQO1 co-expression. A series subgroup analyses was conducted according to clinicopathological characteristics such as gender, smoking history, etc. Results suggested that dual-negative expression of Nrf2 and NQO1 is a prognostic factor for OS in females (*P* = 0.006), non-smokers (*P* = 0.006), and advanced-stage NSCLC patients (stage III–IV) (*P* = 0.023), but no such significance was found in males, smokers, and patients with early stage NSCLC (stage I–II) (Figure 3). In addition, we analyzed Nrf2 and NQO1 expression in the OS of each subgroup. Nrf2 expression predicted a similar OS for subgroups (Supplementary Figure 1). Elevated Nrf2 expression predicted a poor OS in females (*P* = 0.016), nonsmokers (*P* = 0.024), and advanced-stage NSCLC patients (stage III–IV) (*P* = 0.004). NQO1 expression predicted a poor OS in the female subgroup (*P* = 0.029). However, no significance differences were seen in other subgroups (Supplementary Figure 2).

## DISCUSSION

In this study, we detected expression of Nrf2 and NQO1 in tumor tissues of NSCLC patients and investigated the roles of these genes as prognostic factors. Nrf2 expression is elevated in tumors such as gastric cancer [12], colorectal cancer [14], and ovarian epithelial carcinoma [15] and it might correlate with the poor outcomes for patients in these studies. NQO1 is a downstream gene of Nrf2 and is expressed in tumor tissues such as breast cancer [16] and gastric adenocarcinoma [13], indicating a poor outcome in these tumor types. In our study, Nrf2 expression was elevated in nearly all NSCLC tumor tissues compared with the adjacent nontumor tissues, which is consistent with previous results [17]. High NQO1 expression was correlated with high Nrf2 expression. This finding was reasonable because of the connection between Nrf2 and NQO1, as previously mentioned.

We also found that Nrf2 only predicts OS, whereas NQO1 only predicts DFS, of NSCLC patients. Previous studies revealed that Nrf2 might be a factor in tumor initiation and progression [3]. However, Nrf2 has a different function at different tumor stages. Several studies showed that Nrf2 is a tumor suppressor [18], whereas other studies provided evidence that Nrf2 knock-out inhibits tumor growth, and an abnormal state of Nrf2 conferred chemo-resistance [4]. In lung cancer patients, studies on correlation between Nrf2 expression and prognosis also generated paradoxical results. A study by Yang et al. [19] found that Nrf2 expression predicted chemo-resistance and tumor progression in NSCLC patients, whereas Prx1 rather than Nrf2 was considered the independent prognostic factor in another study [17]. Results were similar when NQO1 was considered as an independent prognostic factor. In limited-stage small cell lung cancer, Kim et al. [18] reported no significant correlation between NQO1 and prognosis. The paradoxical conclusion suggested Nrf2 or NQO1 alone might not be a prognostic factor. Thus, reassessing the correlation of Nrf2 and other varieties with prognosis might be useful. As mentioned above, our results indicated that NQO1 expression was positively correlated with Nrf2 expression. Considering that neither Nrf2 nor NQO1 alone is predictive of NSCLC outcomes and their linkage in the context of oxidative stress and tumor progression, we combined Nrf2 and NQO1 as a co-factor in this study and investigated the possibility of their usefulness as prognostic biomarkers in NSCLC patients. Dual-negative expression of Nrf2 and NQO1 suggested Superior OS (*P* = 0.020) and Superior DFS (*P* = 0.037), compared with the mono-expression or co-expression group.

Nrf2 expression was higher in tumors with lymph node (LN) metastasis than in those without metastasis (*P* = 0.001). A similar result was reported in gastric cancer, in which Nrf2 was found to have a correlation with lymphatic invasion [12, 20]. Previous studies found a correlation between a high level of NQO1 and LN metastasis in breast cancer [16] and NSCLCs [21], which was not seen in this study. In addition, good-moderate differentiation was found with a high level of Nrf2 (*P* = 0.032). However, the potential function of Nrf2 in tumor differentiation is yet unclear.

Subgroup analyses suggests dual-negative expression of Nrf2 and NQO1 presents superior outcomes for both OS and DFS in advanced-stage NSCLC patients, rather than in early stage NSCLC patients. That outcome is consistent with previous results reported by other two groups: Kim et al. [17] found Nrf2 was not an independent prognostic factor in stage I NSCLC, and Yang et al. [19] found Nrf2 can be predictive in chemo-resistance and prognosis in stage IIIB or IV NSCLC patients. However, the proportion of cases from different stages in our study was not balanced, because surgery was not generally recommended in patients with stage IV NSCLC. Thus, tumor tissues from this stage were difficult to acquire. Further research might be needed in stage IV patients. In addition, we found that dual-negative expression of Nrf2 and NQO1 appeared to be a prognostic factor for OS in females or nonsmokers, but not in males or smokers. These populations might have commonalities, because most females in China are nonsmokers. No correlation was found between smoking status of patients and Nrf2-NQO1 expression. No similar results were previously reported. Individual Nrf2 expression showed a similar result in OS of the female, nonsmoker, and advanced- stage (III–IV) subgroups. Because the female or nonsmoker subpopulation has an OS outcome like that of the EGFR tyrosine kinase inhibitor (TKI) sensitive population in Asia, we ask whether EGFR stimulates the Nrf2 signaling pathway. Previous studies reported that EGFR stimulates the Nrf2 pathway. On one hand, EGFR promotes Nrf2-activated cell proliferation in lung cancer cells through its downstream MAPK/Erk signaling pathway [22]. On the other hand, nuclear EGFR directly interacts with and phosphorylates nuclear Keap1, a protein that suppresses Nrf2. The nuclear protein level of Keap1 is reduced, resulting in nuclear Nrf2 stabilization and its transcriptional activity increases in cancer cells, which contributes to tumor cell resistance to chemotherapy [23]. Several preclinical or clinical trials are using agents such as sulforaphane and brusatol that activate or inhibit the Nrf2 pathway, respectively [3]. The results from subgroup analyses might provide information about suitable populations in which to study drugs that influence Nrf2 pathways.

## MATERIALS AND METHODS

### Cases

Lung cancer patients who received tumor resection from 2006 to 2011 were selected, and only patients with primary NSCLC tumors were included. Tumor tissues were collected from the Department of Tumor Tissue Bank and the Department of Pathology of Zhejiang Cancer Hospital, China. Clinical and pathological data of patients were collected from the electronic medical record. Survival data were retrospectively analyzed by reviewing medical records or telephone follow-up.

### Ethics statement

The research program was reviewed and approved by the Ethics Committee of Zhejiang Cancer Hospital, and informed consent was obtained from each study subject. We confirmed that all methods were performed in accordance with the relevant guidelines and regulations.

### Immunohistochemical analysis

IHC staining was performed to detect the expression of Nrf2 and NQO1 in tumor tissue. The 5-μm thick tissue sections were de-paraffinized and rehydrated. The antigen was retrieved at 95°C for 20 minutes with Dako EnVisionTM FLEX Target Retrieval Solution (pH 9.0) in the Dako PT Link units. Slides were blocked in 3% hydrogen peroxide for 10 minutes to eliminate endogenous peroxidase activity, and then incubated for 60 minutes at ambient temperature with primary antibodies for Nrf2 (rabbit polyclonal antibody, 1:100, sc-722) (Santa Cruz Biotechnology, Dallas, TX, USA) or NQO1 (mouse monoclonal antibody, 1:100, sc-32793) (Santa Cruz Biotechnology) and incubated in a Dako Envision Flex amplification kit for 30 minutes. Immune complex and nuclei were visualized by incubating the sections with 3, 3′-diaminobenzidine and hematoxylin, respectively.

### Evaluation and scoring

Expression of Nrf2 and NQO1 were assessed based on criterion in a reported study, with some modifications [12]. Both staining intensity and the percentage of positive tumor cells were considered in scoring as follows.

(1) Staining intensity: 0, absence of staining; 1, weak staining; 2, moderate staining; and 3, strong staining.

(2) Positive cells: 0, 0%–25% of staining; 1, 25%–50% staining; 2, 50%–75% staining; 3, ≥75% staining.

Overall scores were obtained by multiplying the scores from (1) and the scores from (2). For Nrf2, the overall score value 9 was defined as high expression. Cases with nuclear Nrf2 immunostaining in more than 10% of tumor cells were regarded as high expression. For NQO1, the overall score value ≥ 6 was defined as high expression.

### Statistical analyses

All statistical analyses were performed using SPSS software for Windows, Version 17.0 (SPSS, Inc., Chicago, IL, USA). The correlation analysis of Nrf2 and NQO1 expression was evaluated by the Pearson Correlation test. Differences of Nrf2 or NQO1 expression in patients with different clinicopathological status were detected by the chi-squared (χ^2^) and Fisher's exact tests. Disease-free survival (DFS) and overall survival (OS) were defined as the time from the surgery date to the cancer recurrence date and date of death for any patient, respectively. If the patient was lost to follow-up, DFS or OS was estimated by use of the date of last follow-up. For survival analysis, the Kaplan-Meier method was used and statistical significance was assessed by use of the log-rank test. Univariate and multivariate analyses were performed by the Cox proportional hazards regression model. For analysis of Nrf2-NQO1 as a co-factor in predicting survival, patients were classified into three groups according to the IHC results: dual-positive (both Nrf2 and NQO1 high expression), mono-positive (either Nrf2 or NQO1 high expression), and dual-negative (both Nrf2 and NQO1 low expression). For subgroup analysis, patients were divided into subgroups according to their clinicopathological characteristics, such as gender and smoking history, and survival analyses were also performed by use of the Kaplan-Meier method. For all tests, *P* < 0.05 was considered statistically significant.

## CONCLUSIONS

Our study suggested that the dual-negative expression of Nrf2 and NQO1 predicts superior survival outcomes in NSCLC patients. Compared with mono-factor Nrf2 or NQO1, the co-factor Nrf2 and NQO1 was more useful in predicting overall survival (*P* = 0.020) and disease-free survival of patients. Females, nonsmokers, and patients with advanced-stage NSCLC may be suitable subpopulations for predicting prognosis by Nrf2-NQO1expression.

## SUPPLEMENTARY MATERIALS FIGURES AND TABLES


