# Supplementary material for: Dual-negative expression of Nrf2 and NQO1 predicts superior outcomes in patients with non-small cell lung cancer
Source: Oncotarget. 2017 Apr 25;8(28):45750–8. doi: 10.18632/oncotarget.17403 (PMC5542223; doi:10.18632/oncotarget.17403)
Supplement: Supplementary file 1 [file oncotarget-08-45750-s001.pdf]

Dual-negative expression of Nrf2 and NQO1 predicts superior outcomes in patients with non-small cell lung cancer

SUPPLEMENTARY MATERIALS

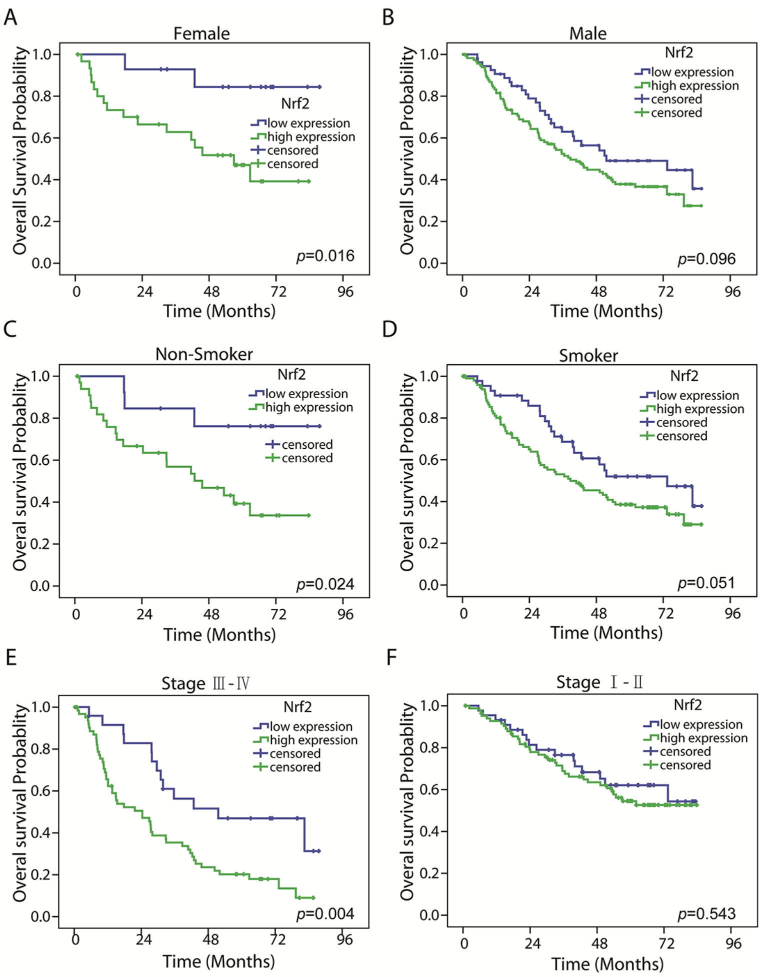

Supplementary Figure 1: Subgroup analyses of Nrf2 in predicting OS. Kaplan-Meier survival analysis estimates the predictive role of Nrf2 by overall survival rates in female **(A)**, male **(B)**, nonsmoker **(C)**, smoker **(D)**, advanced-stage NSCLC **(E),** and early stage NSCLC **(F)** populations. The statistical significance was assessed using the log-rank test.

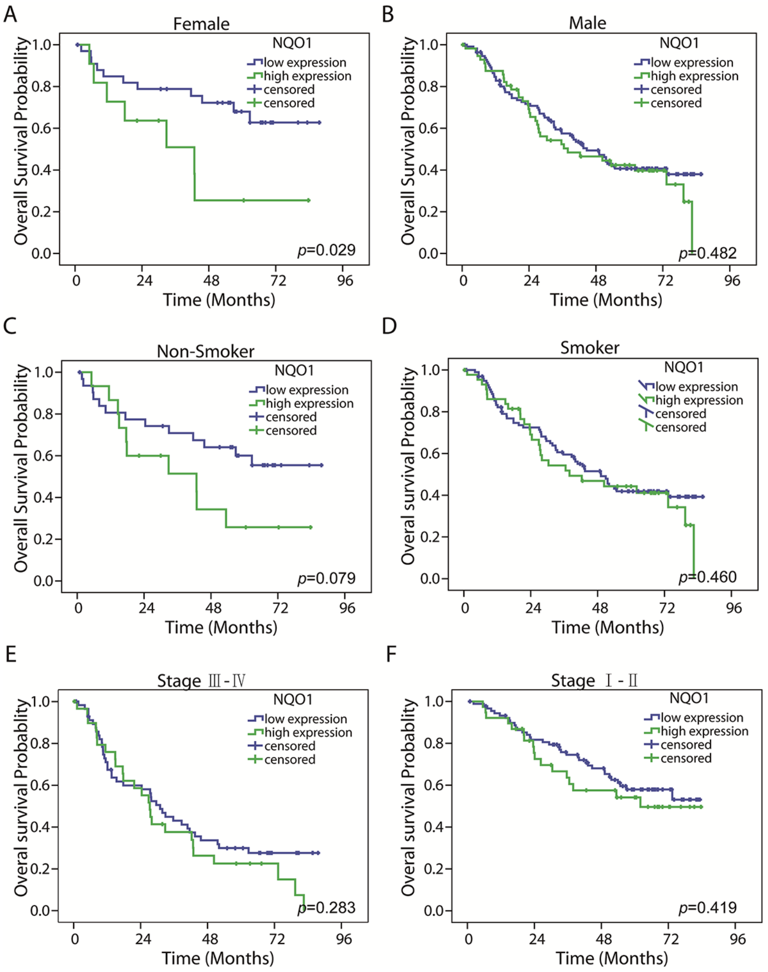

Supplementary Figure 2: Subgroup analyses of NQO1 in predicting OS. Kaplan-Meier survival analysis estimates the predictive role of NQO1 by overall survival rates in female **(A)**, male **(B)**, nonsmoker **(C)**, smoker **(D)**, advanced-stage NSCLC **(E),** and early stage NSCLC **(F)** populations. The statistical significance was assessed using the log-rank test.

Supplementary Table 1: The correlation between Nrf2 and NQO1 expression status

	NQO1			***p***
	**Low**	**High**	**Total**	
Nrf2				0.024
Low	55	13	68	
High	92	55	147	
Total	147	68	215	

The correlation between NQO1 and Nrf2 expression was evaluated by Pearson correlation test.
